# Changes to Saccade Behaviors in Parkinson’s Disease Following Dancing and Observation of Dancing

**DOI:** 10.3389/fneur.2013.00022

**Published:** 2013-03-11

**Authors:** Ian G. M. Cameron, Donald C. Brien, Kira Links, Sarah Robichaud, Jennifer D. Ryan, Douglas P. Munoz, Tiffany W. Chow

**Affiliations:** ^1^Helen Wills Neuroscience Institute, University of California BerkeleyBerkeley, CA, USA; ^2^Centre for Neuroscience Studies, Queen’s UniversityKingston, ON, Canada; ^3^Baycrest Rotman Research InstituteToronto, ON, Canada; ^4^Dancing with Parkinson’s Inc.Toronto, ON, Canada; ^5^Department of Biomedical and Molecular Science, Queen’s UniversityKingston, ON, Canada; ^6^Department of Psychology, Queen’s UniversityKingston, ON, Canada; ^7^Department of Medicine, Queen’s UniversityKingston, ON, Canada; ^8^Department of Medicine, University of TorontoToronto, ON, Canada; ^9^Department of Psychiatry, University of TorontoToronto, ON, Canada

**Keywords:** action observation, anti-saccade, basal ganglia, dance, Parkinson’s disease, pro-saccade

## Abstract

**Background:** The traditional view of Parkinson’s disease (PD) as a motor disorder only treated by dopaminergic medications is now shifting to include non-pharmacologic interventions. We have noticed that patients with PD obtain an immediate, short-lasting benefit to mobility by the end of a dance class, suggesting some mechanism by which dancing reduces bradykinetic symptoms. We have also found that patients with PD are unimpaired at initiating highly automatic eye movements to visual stimuli (pro-saccades) but are impaired at generating willful eye movements away from visual stimuli (anti-saccades). We hypothesized that the mechanisms by which a dance class improves movement initiation may generalize to the brain networks impacted in PD (frontal lobe and basal ganglia, BG), and thus could be assessed objectively by measuring eye movements, which rely on the same neural circuitry.

**Methods:** Participants with PD performed pro- and anti-saccades before, and after, a dance class. “Before” and “after” saccade performance measurements were compared. These measurements were then contrasted with a control condition (observing a dance class in a video), and with older and younger adult populations, who rested for an hour between measurements.

**Results:** We found an improvement in anti-saccade performance following the observation of dance (but not following dancing), but we found a detriment in pro-saccade performance following dancing.

**Conclusion:** We suggest that observation of dance induced plasticity changes in frontal-BG networks that are important for executive control. Dancing, in contrast, increased voluntary movement signals that benefited mobility, but interfered with the automaticity of efficient pro-saccade execution.

## Introduction

It has recently been shown that regular exercise and rhythmic movements (e.g., bike riding, dancing) can improve bradykinetic symptoms in Parkinson’s disease (PD) (Goodwin et al., 2008; Earhart, 2009; Hackney and Earhart, 2009). Similarly, people with PD can make performance gains on one cognitive task (Stroop: name the print color rather than read a written word) after training on another (Sudoku: place numbers into a grid in a mathematically constrained pattern) (Nombela et al., 2011). It is possible that plasticity changes from learning to perform these tasks induces general alterations to the fronto-basal ganglia (BG) circuits affected by PD (Betchen and Kaplitt, 2003; Rodriguez-Oroz et al., 2009; Leh et al., 2010; Beeler, 2011). What is unknown however, is how long these changes can last, and how widely their effects on other cognitive or motor functions can map.

We have noticed immediate but short-lasting changes to PD patients’ mobility after participating in a 1-h dance class (Sarah Robichaud and Tiffany W. Chow, unpublished observations, www.dancingwithparkinsons.com/press.html). This raises an important question as to whether such beneficial changes could translate to improved performance on other tasks assessed immediately after dancing. In particular, could improvement in voluntary movement generation be objectively measured with eye tracking, given that the saccadic eye movement system taps into well-characterized deficits in motor function in PD? (Munoz and Everling, 2004). Therefore, participants with PD performed pro- and anti-saccades before and after a dance class. Pro-saccades (look to a visual target) were examined because they are highly automatic movements previously shown *not* to be impaired in PD (Chan et al., 2005; Cameron et al., 2010, 2012). In contrast, anti-saccades (look in the opposite direction) have consistently been shown to be impaired in PD, both at the executive level (greater direction errors) and at the motor level, such that they are executed with longer latencies and are often hypometric (Briand et al., 1999; Chan et al., 2005; Mosimann et al., 2005; Amador et al., 2006; Hood et al., 2007; Rivaud-Pechoux et al., 2007).

We compared pro- and anti-saccade behavior following the dance class to baseline behavior assessed immediately prior to the dance class. The same procedure was replicated when participants watched a video of their dance class, instead of dancing. It has recently been shown that pathological beta-band oscillations in the subthalamic nucleus (STN) of the BG decrease when PD patients observe as well as perform wrist rotations, implicating the human “mirror neuron” system whereby the brain codes action observation similar to action execution (Alegre et al., 2010).

Taken together, we develop the following hypotheses. First, dancing is a complex activity demanding voluntary motor control and learning that engages fronto-BG networks; therefore, plasticity benefits can lead to subsequent improvements in saccade tasks. Second, there should be larger improvements to anti-saccade performance, because anti-saccades require greater voluntary control and have previously been shown to be impaired in PD. Finally, dancing should result in larger improvements compared to video observation, because it actively engages the fronto-subcortical motor networks. However, some improvements may be observed following video observation, perhaps due to the mirror neuron system.

## Materials and Methods

Twenty-three people with PD (age range 63–88 years, mean 74 years; 14 males) were recruited from a group of people actively participating in a weekly dance class run by co-author Sarah Robichaud in Toronto, ON, Canada. The instructors had created a DVD of a dance class that was then used in a “watch only” video intervention. Participants were requested to attend two sessions at the Rotman Research Institute, Toronto, whereby clinical assessment of the stage of illness was recorded as Hoehn and Yahr scores (Hoehn and Yahr, 1967) by two neurologists (Tiffany W. Chow and Silvia Rios-Romenets) and a trained research assistant (Kira Links). Twenty-one healthy older adults (range 64–83, mean 72; 8 males) and 19 healthy younger adults (range 20–23, mean 21; 8 males) were recruited from the Rotman Research Institute, and Queen’s University, Kingston, ON, Canada communities, respectively, in order to determine if there were any basic practice effects from performing the tasks in two sessions only 1 h apart (see below). We refer to these populations as “controls,” noting, however, that they did not participate in weekly dance classes, nor receive the dance and video intervention, but served only to control for a practice effect. PD patients and healthy older adults performed the eye movement tasks at the Rotman Research Institute, and healthy younger adults performed the eye movement tasks at Queen’s University, Kingston, ON, Canada using the same methods and eye tracking equipment. The study was approved by the Research Ethics Boards of the Rotman Research Institute and Queen’s University, and adhered to the Canadian Tri-Council Policy on the Conduction of Research Involving Humans and to the Declaration of Helsinki. Participants were compensated for their transportation and time.

Parkinson’s disease patients were tested on two separate days. On the first day all participants were tested in the eye tracking task. Following this, half the participants were randomly assigned to the dance class group, and the other half were assigned to the video group. After this dance or video intervention, every participant was re-tested in the same order as initially. One month later, this same procedure was repeated, but with the participants assigned to the opposite intervention. In the control groups, participants were assessed before and after a 1-h “rest” intervention on 1 day only.

Two portable ISCAN (Burlington, MA, USA), two portable Eyelink 1000, and two stationary Eyelink II (SR Research, Mississauga, ON, Canada) eye tracking systems were utilized to assess saccade behavior using head-mounted infrared cameras. The right eye was tracked. Eye movements were recorded at 500 Hz on Eyelink and 250 Hz on ISCAN systems, with participants sitting approximately 57 cm from LCD computer monitors (19′′). The timing of trial events was identical across all systems, and stimuli were matched for size, eccentricity, and luminance.

**Figure 1:**
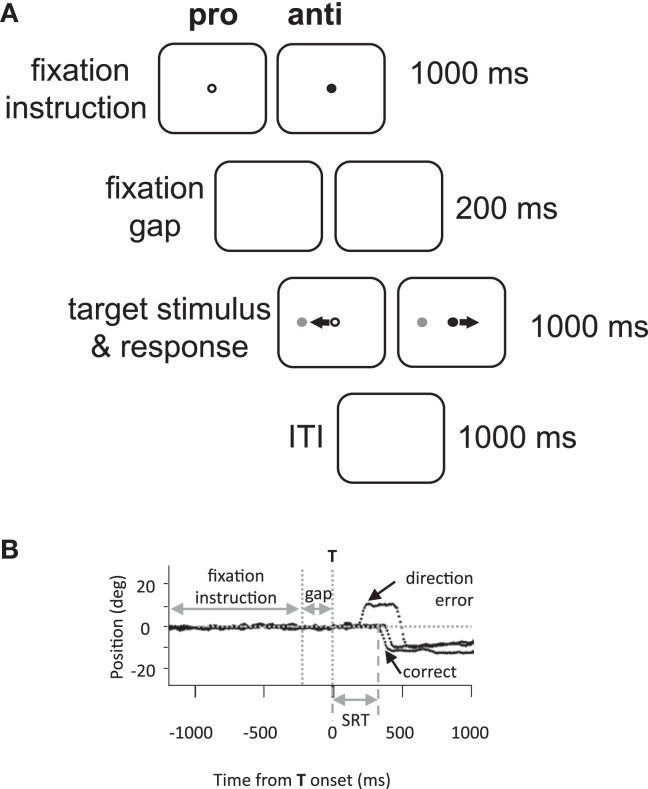
**Methods**. **(A)** Experimental paradigm and timings of events. Anti, anti-saccade; ITI, inter-trial interval; pro, pro-saccade. **(B)** Illustration of eye position traces for correct leftward anti-saccade trials, and a direction error on an anti-saccade trial. SRT, saccade reaction time; T, target stimulus.

Eye movement assessment consisted of 60 pro- and 60 anti-saccade trials (30 leftwards and 30 rightwards of each) in an interleaved and pseudo-random presentation. Each trial was 3.2 s in duration, and thus each 120 trial assessment lasted 6.4 min. A green or red central fixation point was illuminated on a black background for 1000 ms (Figure 1A). After a delay of 200 ms following the disappearance of the fixation point, a gray stimulus (“target”) appeared at 10° to the left or right of center. Participants were required to initiate and complete a saccade based on fixation point color. In the pro-saccade task (green fixation), participants were instructed to look toward the target as soon as it appeared; while in the anti-saccade task (red), participants were instructed to look to the mirror location on the opposite side. Participants were given 1000 ms to complete the saccade, and an inter-trial interval of 1000 ms (blank screen) followed.

Saccades were analyzed offline by custom MATLAB (MathWorks, Natick MA, USA) programs. Saccades were defined as the first deflection by >3 SDs over the background mean velocity following target onset. The behavioral parameters of interest were designated as follows: saccadic reaction time (SRT) as the time from target onset to the initiation of the first saccade; the coefficient of intra-subject variability in SRT (CV) as the standard deviation of a participant’s SRT distribution divided by the mean; direction errors as any initial saccade initiated in the wrong direction (e.g., Figure 1B); anticipatory errors as saccades with SRT <90 ms (Munoz et al., 1998), and saccade amplitude in degrees.

Statistical analyses were performed in MATLAB. We first performed a four-way ANOVA with the factors Time (before and after), Task (pro-saccade or anti-saccade), Intervention (dance or video), and Day (1 or 2). Repeated measures ANOVAs were not performed, because there were four Day-1 participants who did not return on Day-2, and two new participants were added in their place. The four who left had received the dancing intervention on Day-1, and one of the two new participants on Day-2 received dance. Two-way ANOVAs were also performed for PD participants for each task separately to look for significant Time × Intervention interactions. For control populations, two-way ANOVAs were performed with the factors Time and Task. Repeated measures ANOVAs were not performed in controls to maintain consistency with the PD participants.

Our main *a priori* tests of interest consisted of paired *t*-tests to contrast before versus after measures (from the Time factor), separately for the video and dance intervention in the PD group, and separately for each task and each group. Also, independent *t*-tests were performed across interventions for identical tasks for the “before” Time factor (e.g., video-anti-before compared to dance-anti-before), to determine if there were any differences in the data despite the random assignment of PD participants to the intervention groups. Hedges’ *g* values are provided as measures of effect sizes. Because we hypothesized that we would be assessing temporary improvements in saccade behaviors, we made sure to test the saccades immediately after the dance or video intervention, rather than re-perform a clinical assessment.

## Results

Overall, results show that pro-saccade behavior deteriorated following dancing, but anti-saccade behavior improved following the video observation. These patterns in behavioral changes (following the dance or video intervention in PD) were not the same as those observed in the control groups after the rest period. Our main interest was in SRT and percentage direction errors, as they are the most important parameters reflecting changes in the control over voluntary movement. However, we also describe reaction time variability (CV), saccade amplitude, and percentage anticipatory errors, as they are also common parameters for describing saccade behavior. Statistical significance was taken at *P* < 0.05.

### Participants with PD

**Figure 2:**
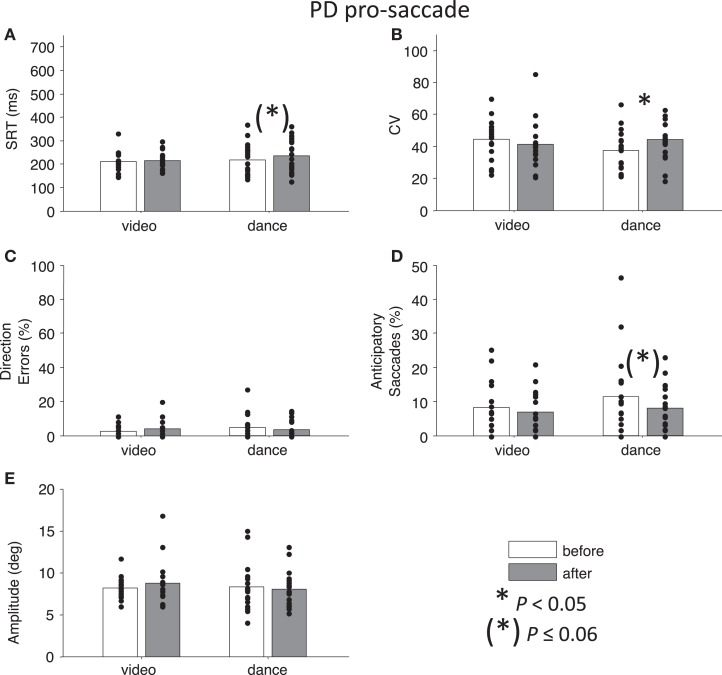
**Pro-saccade behavior in participants with PD**. **(A)** Saccade reaction time (SRT). **(B)** Intra-subject coefficient of variability in saccade reaction time (CV). **(C)** Percentage direction errors. **(D)** Percentage anticipatory saccades. **(E)** Saccade amplitude. Dots indicate each participant. Asterisks indicate significant differences between before and after measurements (paired *t*-tests, *P* < 0.05).

**Figure 3:**
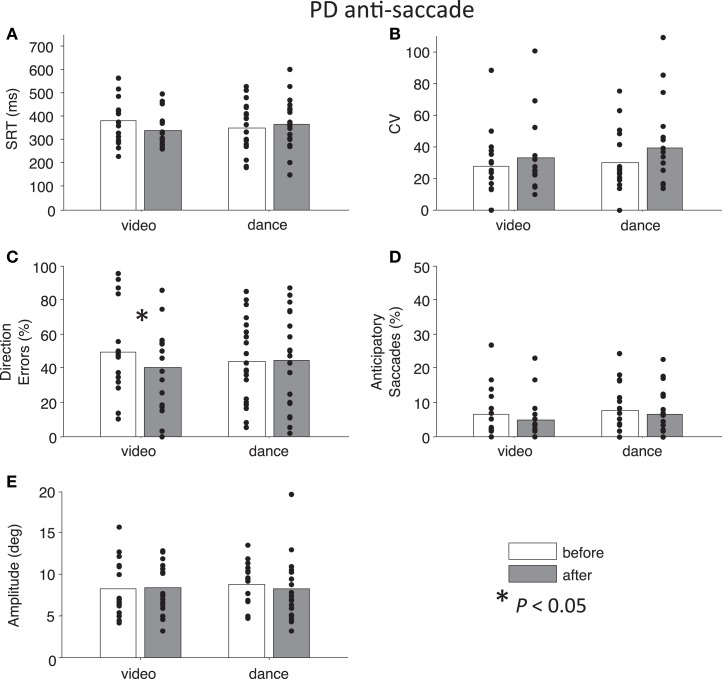
**Anti-saccade behavior in participants with PD**. **(A)** Saccade reaction time (SRT). **(B)** Intra-subject coefficient of variability in saccade reaction time (CV). **(C)** Percentage direction errors. **(D)** Percentage anticipatory saccades. **(E)** Saccade amplitude.

First, we conducted a four-way ANOVA across each of the behavioral parameters. For SRT, there was an Intervention × Day interaction that approached significance, *F*(1,127) = 3.73, *P* = 0.06. Likewise, for percentage direction errors, there was an Intervention × Day interaction that approached significance, *F*(1,128) = 3.76, *P* = 0.055, and there was also a main effect of Task, *F*(1,128) = 162.28, *P* < 0.01. For CV, there was a significant Time × Task × Day interaction *F*(1,127) = 4.97, *P* < 0.05, and there was a significant main effect of Task, *F*(1,127) = 9.77, *P* < 0.01. For percentage anticipatory saccades, the main effect of Task was marginally significant *F*(1,128) = 3.24, *P* = 0.07. Finally, there were no significant interactions or main effects for saccade amplitude, *P*s > 0.54. Overall, the main effects of Task are illustrated in Figures 2 and 3: pro-saccades were easier to perform for PD participants, but were associated with more variable SRT. Note that the day the participants received our primary manipulation of interest (Intervention) was counterbalanced, so Day was not examined further. From the two-way ANOVAs run separately for each task, no statistical tests reached significance (all *P*s > 0.13).

Next, we addressed our *a priori* interests by assessing Time separately for each Intervention factor. Paired *t*-tests showed that pro-saccade SRTs were marginally *longer* following the dance intervention *t*(19) = −2.04, *P* = 0.06, *g* = −0.27, while there was no effect of Time for the video intervention, *P* = 0.76 (Figure 2A). For anti-saccades, there was no significant differences in SRT for either intervention, *P* > 0.09. The CV, a measure of how variable a participant’s SRT is, was significantly *increased* for pro-saccades following dancing, *t*(19) = −2.85, *P* < 0.05, *g* = −0.55 (Figure 2B), and no other test involving CV approached significance, *P* > 0.17. For pro-saccades, there was no significant difference in direction errors for either intervention *P* > 0.23 (Figure 2C), however, the video intervention significantly *reduced* direction errors for anti-saccades, *t*(15) = 2.6, *P* < 0.05, *g* = 0.33, but dance did not, *t*(19) = −0.23, *P* = 0.8 (Figure 3C). A reduction in the percentage of anticipatory saccades for pro-saccades following dance approached significance, *t*(19) = 2.08, *P* = 0.05, *g* = 0.37 (Figure 2D). Finally, there were no changes in saccade amplitude for pro- or anti-saccades after either intervention (*P* > 0.40) (Figures 2E and 3E), and no other tests approached significance, *P* > 0.19.

To address the possibility that there were differences in baseline behavior between the Intervention groups that could have impacted the intervention effects, independent *t*-tests were performed: there were no baseline differences in SRT (*P*s > 0.30), CV (*P*s > 0.11), percentage direction errors (*P*s > 0.18), percentage anticipatory errors (*P*s > 0.35), or amplitude (*P*s > 0.40), between the Interventions for identical Tasks in the before condition.

**Figure 4:**
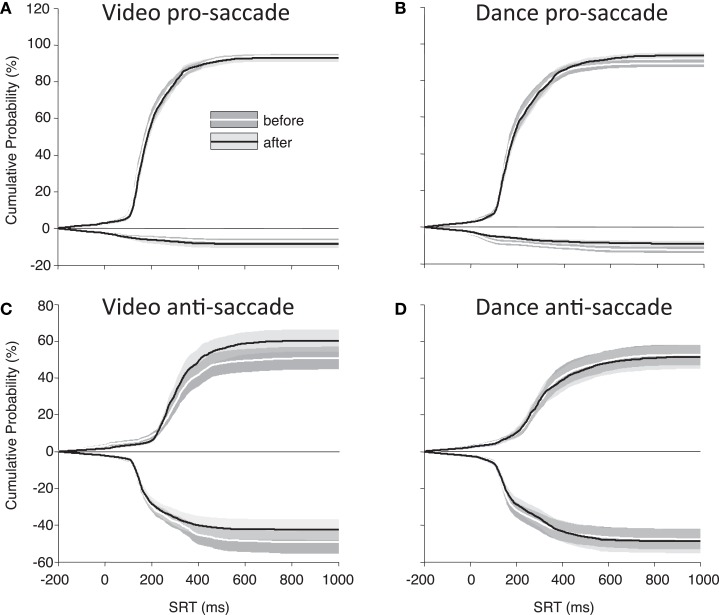
**Cumulative percent probabilities in SRT for participants with PD**. **(A)** Pro-saccade, video; **(B)** pro-saccade, dance; **(C)** anti-saccade, video; **(D)** anti-saccades, dance. Positive *Y* values indicate correct saccades, and negative *Y* values indicate direction errors. SRT (*X* axis) begins 200 ms before target onset to include anticipatory saccades. Shading indicates standard error.

Finally, to best illustrate differences in our main parameters of interest (direction errors and SRT), Figure 4 shows the cumulative distributions of SRT for the PD participants as a group. These plots illustrate whether participants have made more, or fewer, correct responses for a given SRT value. There is a noticeable improvement in anti-saccade performance following the video intervention (Figure 4C), and there are increases in correct pro-saccades following the dance intervention (Figure 4B).

### Correlations with disease stage in PD

Pearson (*r*) correlations were performed between Hoehn and Yahr scores and the difference (after − before) values for each of the five behavioral parameters of interest, to examine whether any changes due the video or dance intervention were correlated with disease stage. There were no significant correlations with SRT (all *P*s > 0.14). However, there was a significant negative correlation with the difference in CV on pro-trials for the dance intervention, *r* = −0.52, *P* < 0.05, indicating that higher Hoehn and Yahr scores were associated with less of an increase in CV following the dance intervention in pro-saccades. There was a negative correlation that approached significance with the reduction in percentage direction errors on pro-trials for the dance intervention, *r* = −0.49, *P* = 0.05; however, there was a significant positive correlation between Hoehn and Yahr score, and the increase in percentage direction errors on pro-trials for the video intervention, *r* = 0.61, *P* < 0.05. There was a marginally significant negative correlation between Hoehn and Yahr score, and the reduction in percentage anticipatory saccades on anti-trials for the video intervention, *r* = −0.47, *P* = 0.07. Finally, there were no significant correlations with amplitude (all *P*s > 0.33x), and no other correlations involving Hoehn and Yahr score approached significance (all *P*s > 0.14).

Marginally significant negative correlations of *age* also occurred with fewer anticipatory errors, following dancing, but not video, for both pro-saccades, *r* = −0.49, *P* = 0.06 and anti-saccades, *r* = −0.46, *P* = 0.07. No other correlations with age approached significance (all *P*s > 0.15).

In summary, correlations suggests that dancing seemed to have worse effects on pro-saccade performance in patients who were less advanced in disease state, whereas watching the video seemed to have beneficial effects on anti-saccade behavior in patients who were more advanced.

### Control participants

**Figure 5:**
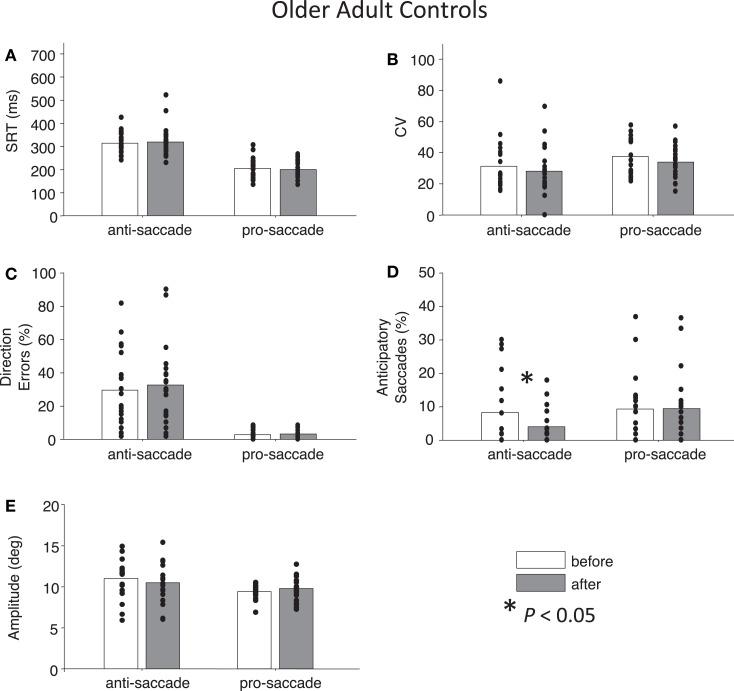
**Anti- and pro-saccade behavior in healthy older adults**. Horizontal axis now indicates Task (pro- or anti-saccade) and legend indicates the Time factor (before or after a period of rest). **(A)** Saccade reaction time (SRT). **(B)** Intra-subject coefficient of variability in saccade reaction time (CV). **(C)** Percentage direction errors. **(D)** Percentage anticipatory saccades. **(E)** Saccade amplitude.

**Figure 6:**
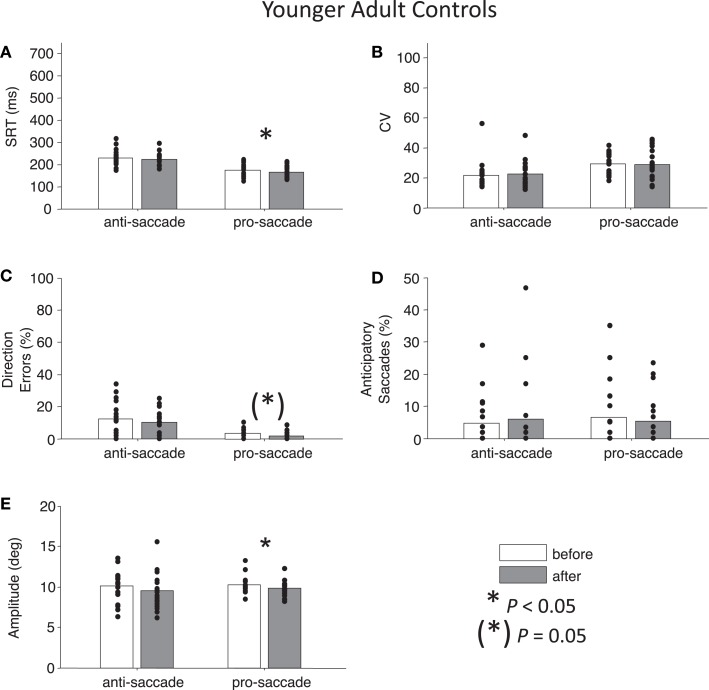
**Anti- and pro-saccade behavior in healthy younger adults**. **(A)** Saccade reaction time (SRT). **(B)** Intra-subject coefficient of variability in saccade reaction time (CV). **(C)** Percentage direction errors. **(D)** Percentage anticipatory saccades. **(E)** Saccade amplitude.

For both control groups, there were no interactions between Time and Task for any the factors as assessed by two-way ANOVAs (all *P*s > 0.28), indicating that “rest” did not have different effects on anti- or pro-saccades. As expected, older controls showed significant main effects of Task for SRT, *F*(1,76) = 96.01, *P* < 0.01 and for percentage direction errors, *F*(1,76) = 55.82, *P* < 0.01, and also a marginal effect of Task for CV, *F*(1,76) = 3.7, *P* = 0.06 (Figures 5A–C). There was no significant main effect of Task for anticipatory saccades (*P* > 0.12) (Figure 5D), but there was for amplitude, *F*(1,76) = 7.38, *P* = 0.01, indicating that anti-saccades were of greater amplitude than pro-saccades (Figure 5E). The results for young adults were similar: significant main effects of Task occurred for SRT, *F*(1,72) = 67.77, *P* < 0.01, percentage direction errors, *F*(1,72) = 31.54, *P* < 0.01, CV, *F*(1,72) = 12.79, *P* < 0.01, but not anticipatory saccades (*P* > 0.78) (Figures 6A–D). However, there were no significant main effects or interactions for amplitude (*P* > 0.21) (Figure 6E).

While neither control group showed any significant main effects of Time (all *P*s > 0.27), paired *t*-tests did reveal a significant reduction in percentage anticipatory saccades for anti-saccades in the older adults, *t*(19) = 2.51, *P* < 0.05, *g* = 0.33 (Figure 5D). There were no other significant differences across the other parameters for either task in older adult controls (all *P*s > 0.18). For young adult controls, there was a significant reduction in SRT for pro-saccades *t*(18) = 2.11, *P* < 0.05, *g* = 0.29, but not for anti-saccades (*P* = 0.09) (Figure 6A). The was also a marginally significant reduction in percentage direction errors for pro-saccades, *t*(18) = 2.04, *g* = 0.50, *P* = 0.05, but not for anti-saccades (*P* = 0.30), Figure 6C. No differences were found for CV or percentage anticipatory saccades for either task (all *P*s > 0.34). Finally, there were no significant differences in amplitude for healthy older adults for either task (*P* > 0.29) (Figure 5E), however there were significant reductions in amplitude for pro-saccades in young adults, *t*(18) = 2.39, *P* < 0.05, *g* = 0.44, and no differences for anti-saccades (*P* > 0.15) (Figure 6E).

### Main summary

After the dance class, participants with PD were slower and more variable in their pro-saccade responses, though the percentage of anticipatory pro-saccades was reduced. Conversely, after watching the dance video, the number of direction errors on anti-saccade trials was significantly reduced. Such effects were not present in the control participants who only received a “rest” intervention.

## Discussion

We designed an experiment to determine if the reduced bradykinetic symptoms we observed in PD following dancing could be measured by improvements in saccade control. We hypothesized that any behavioral changes after the intervention would indicate plasticity changes in fronto-BG circuitry. Actual changes in plasticity are impossible to determine in the current study, as with many studies of human behavior (though Transcranial Magnetic Stimulation has provided evidence that changes in motor-system plasticity do occur in a number of human studies, e.g., O’Shea et al., 2007; Kojovic et al., 2012). Instead, we characterized parameters from saccade tasks that would indicate improvements in fronto-BG signaling important to voluntary control, and took these as a proxy for changes in plasticity.

Interestingly, we observed that watching a dance video improved performance on anti-saccades in PD, whereas dancing itself adversely affected pro-saccade generation (making then marginally slower and more variable in reaction time). To understand how these findings could be produced, it is important to consider that improved anti-saccade behavior can be at the “expense” of pro-saccade behavior, when participants evoke voluntary control over their saccades (Cherkasova et al., 2002; Cameron et al., 2007, 2010; Ethridge et al., 2009). As explained in the following sections, both point to improved voluntary movement control.

The video intervention resulted in reduced direction errors on anti-saccade trials, which is a critical indicator of improved voluntary control over behavior (Figure 3C). Correlation analysis showed that PD patients at a greater stage of the disease actually made more anti-saccades *in error* on pro-saccade trials, and were better at waiting to respond when presented with an anti-saccade instruction. This suggests that the video manipulation improved executive control.

In contrast, marginally increased pro-saccade SRT and increased intra-subject variability in reaction time (CV), but reduced anticipatory errors, following the dance class, suggest that participants had increased their voluntary control over “automatic” pro-saccades. Patients with higher Hoehn and Yahr scores also showed less of an increase in pro-saccade CV, and actually a smaller percentage of direction errors for pro-saccades (though errors were very few). We suggest that these greater stage participants were not as actively in control of their pro-saccades through voluntary means, given the known pathology of PD and its influence on voluntary movement (Mink, 1996; Betchen and Kaplitt, 2003; Nambu, 2005). Thus, our results do demonstrate a beneficial effect of dance on voluntary movement generation. However, benefits of dancing did not translate into the eye movement system in the way we predicted; it adversely affected movements that would ordinarily be produced more automatically. This finding points to the dual role of the BG in action initiation and action suppression, explained below.

### Influence of dance on voluntary movement

A fundamental hypothesis of BG function is that it assists in the suppression of unwanted movements and in the boosting of wanted movements; rigidity in PD, therefore, may relate to “noise” from competing signals not properly filtered by this mechanism (Mink, 1996). The BG are also important for *automatization* – which relates to ability of responses to become initiated with less cortical involvement (Doyon, 2008). Automatization is a process of learning that depends on dopamine to modulate signaling in the BG (Beeler, 2011) and PD patients do have difficulty in achieving automatization (Wu and Hallett, 2005). So how can PD patients be unimpaired on “automatic” pro-saccades?

Pro-saccades are highly automatic to the degree that they are can be executed *without* top-down control from frontal cortex (Munoz and Everling, 2004). The presence of top-down control almost certainly increases pro-saccade latency, as we have shown in more difficult paradigms where pro and anti-saccades are interleaved with task-switch trials (Cameron et al., 2007, 2010). Therefore, we examined another saccade behavioral parameter, “express saccades,” which correspond to highly automatic saccades (latencies typically 90–140 ms) evoked by visual signals that synapse on visuomotor neurons in the superior colliculus (SC) to drive a saccade immediately (Dorris et al., 1997). Their occurrence indicates the absence of top-down inhibition on a given trial, and we have observed an increased frequency of express saccades in PD (Chan et al., 2005; Cameron et al., 2012). Here, paired *t*-tests revealed a significant *decrease* in percentage express saccades on pro-saccade trials following the video intervention, *t*(15) = 2.78, *P* < 0.05, but not dance intervention (*P* = 0.22). This shows that there was greater inhibitory control after the video intervention, and also indicates that the increase in pro-saccade CV following dancing was related to pro-saccades becoming even *less* automatic (i.e., because of a greater number of longer-latency saccades). Based on the fact that dancing delayed pro-saccade execution, but did not reduce direction errors on anti-saccade trials, we suggest that dancing boosted BG signals related to cortical motor execution, thereby interfering with the automaticity of pro-saccades.

An alternative possibility is that dancing may have had a beneficial effect on BG-SC inhibition. The finding that PD patients can exhibit faster pro-saccade SRT and increased express saccades compared to control subjects has been difficult to explain, because while shorter pro-saccade SRT fits with reduced top-down inhibition from frontal cortex, increased inhibition from the BG on the SC should increase latencies. Terao et al. (2011), hypothesized that the while BG-SC inhibition may be increased in PD, it is often “leaky,” in that it may be reduced on a subset of trials leading to an overall decrease in mean saccade latency to visual targets. (Leakiness may relate to pathological oscillations in BG circuits). Thus, it is also possible that the beneficial effect of dancing may have been to reduce this leakiness, resulting in greater mean pro-saccade latency.

### Influence of action observation

How the video intervention improved anti-saccade performance in PD is not clear, though it did seem to have a particular benefit on *executive* control signals, as error rates on anti-saccade trials were reduced. One possibility is that improvement in anti-saccade performance was related to the engagement of the “mirror neuron” system (during the video observation), whereby the brain codes action observation similarly to action–execution (Rizzolatti and Craighero, 2004; Keysers, 2009). The link between the oculomotor system and the mirror system has been established by studies of predictive eye movements in manual tasks (Flanagan and Johansson, 2003; Elsner et al., 2013). Importantly, action observation does not fully engage the execution system, and we suggest it requires executive control against movement execution, given that the patients were watching people perform movements they had been used to performing previously. The main link between inhibitory control and the mirror system may be the inferior frontal gyrus (IFG).

The IFG is an important part of the human mirror neuron system, and is also important to the inhibition of actions (Aron and Poldrack, 2006; Aron et al., 2007; Rowe and Siebner, 2012). The posterior IFG may be more appropriately considered to be part of the human mirror system, as revealed by a recent fMRI study, though the anterior IFG also shows activation patterns consistent with action observation (Press et al., 2012). Little attention has been paid to the IFG in saccade tasks however, because the dorsolateral prefrontal cortex is typically associated with executive control on anti-saccade trials (Munoz and Everling, 2004), and few studies directly examine the IFG. However, it was shown that a patient with an IFG lesion had increased direction errors on anti-saccade trials (Walker et al., 1998), and we have observed greater fMRI activation in the IFG in PD patients when preparing for an anti-saccade compared to preparing for a pro-saccade (Cameron et al., 2012, Table 3), suggesting it has an role in top-down inhibition in the oculomotor system. Finally, recordings from the STN in PD have shown evidence that the BG is also part of the human mirror neuron system (Alegre et al., 2010). This is of particular relevance, because the STN forms part of a “hyperdirect” pathway (Mink, 1996; Nambu, 2004, 2005), which allows the BG to translate executive signals related to stopping/preventing actions from the IFG and the pre-supplementary motor area (Aron and Poldrack, 2006; Aron et al., 2007). Thus, it is possible that video observation lead to improved performance on executive components of the anti-saccade task because participants may have been actively inhibiting movement. We speculate that this executive control may have had a generalized effect on inhibiting automatic movements in the saccade tasks performed immediately after.

### Study limitations

Our goal was to quantify improvements in voluntary motor control using the saccade system that we hypothesized would be temporary; this required us to focus on assessing the PD patients as quickly as possible using the available eye tracking equipment. Ideally, we would have also included other assessments of motor function before and after the interventions, such as the Unified Parkinson’s Disease Rating Scale (UPDRS). However, this assessment has poorer (subjective) reliability compared to eye tracking, and would have extended the post intervention time window. Therefore, while our results do demonstrate how voluntary movement generation is changed after dancing (and observation of dancing), we acknowledge that direct conclusions should only be made with respect to the saccade system. Nevertheless, the saccade system has consistently been shown to be an appropriate proxy for understanding movement initiation in general, as well as the voluntary control over movement, due to the common brain regions and coupling between the manual, limb, and oculomotor systems in orienting (Munoz and Everling, 2004; Munoz and Coe, 2011).

### Implications and conclusion

The results from this study point to an interesting methodological problem: how should one quantitatively measure immediate improvement from dancing in PD? Our use of the saccade system offered indirect evidence of the beneficial effects of dancing on voluntary movement generation. Namely we showed how performing voluntary dance movements adversely affected performance on a task that is normally more automatic. However, the surprising finding that action observation improved parameters related to executive control suggests that future work is needed to understand the mirror system and its relation to executive control, and to select the most appropriate assessments of the generalized benefits to PD from dancing, bike riding or other forms of exercise (Tan, 2007).

## Conflict of Interest Statement

Ian G. M. Cameron, Donald C. Brien, Kira Links, Jennifer D. Ryan, Douglas P. Munoz, and Tiffany W. Chow declare no conflict of interest. Sarah Robichaud is the founder and brand owner of a charity (Dancing with Parkinson’s Inc.) that was involved in recruiting the participants.
